# Is Neoadjuvant Chemoimmunotherapy Safe in Surgically High-Risk Lung Cancer Patients? A Single-Centre IPTW Analysis

**DOI:** 10.3390/jcm15156069

**Published:** 2026-08-04

**Authors:** Fathima Shafra Mubarak, Muneeb Khalid, Jose Alvarez Gallesio, Marco Nardini, Joshil Lodhia, Elaine Teh, Nilanjan Chaudhuri, Kostas Papagiannopoulos, Richard Milton, Alessandro Brunelli, Peter Tcherveniakov

**Affiliations:** Department of Thoracic Surgery, St James’s University Hospital, Leeds Teaching Hospital NHS Trust, Beckett Street, Leeds LS9 7TF, UK

**Keywords:** lung cancer, neo-adjuvant Chemo-IO, non-small cell lung cancer, high risk MDT

## Abstract

**Background:** Neoadjuvant chemoimmunotherapy is now a standard approach for resectable stage II–III non-small cell lung cancer (NSCLC), demonstrating improved pathological response and survival. However, its safety in physiologically high-risk surgical patients is unclear, as these groups are underrepresented in trials. This study compares 30-day and 90-day mortality and oncological outcomes of neoadjuvant chemoimmunotherapy followed by surgery versus upfront surgery within a high-risk multidisciplinary team (MDT) cohort. **Methods:** A retrospective single-centre study was performed, including consecutive high-risk MDT patients undergoing resection for stage II -III NSCLC between January 2022 and December 2025. High-risk status was defined by established physiological, cardiopulmonary, and comorbidity criteria. Patients were stratified into neoadjuvant chemoimmunotherapy followed by surgery (Chemo-IO; *n* = 33) or upfront surgery (*n* = 57). The primary outcome was 30-day mortality. Secondary outcomes included 90-day mortality, R0 resection rate, and length of hospital stay. Inverse probability of treatment weighting (IPTW) based on propensity scores was used to balance baseline differences between groups. **Results:** Ninety patients were included. Patients receiving neoadjuvant therapy had a greater comorbidity burden (Charlson Comorbidity Index 3.2 ± 1.5 vs. 1.4 ± 1.7, *p* < 0.001) and more advanced disease (stage III: 57.6% vs. 29.8%, *p* = 0.014). Thirty-day mortality was 0% in the Chemo-IO group and 1.8% following upfront surgery. After IPTW adjustment, neoadjuvant therapy was not associated with increased perioperative mortality. Ninety-day mortality was similar between groups (3.0% vs. 3.5%; IPTW OR 0.91, 95% CI 0.05–15.39, *p* = 0.948). R0 resection rates were numerically higher following Chemo-IO (87.9% vs. 78.9%; IPTW OR 3.42, 95% CI 0.71–16.59, *p* = 0.127). Median hospital stay was identical in both cohorts (6 days), with no significant difference after weighting (*p* = 0.722). **Conclusions:** Neoadjuvant chemoimmunotherapy appears safe and feasible in carefully selected high-risk patients with resectable stage II–III NSCLC. Despite greater comorbidity burden and more advanced disease, short-term perioperative outcomes were not compromised, while a trend towards improved R0 resection rates was observed. These findings support the consideration of multimodal treatment strategies within high-risk MDT pathways.

## 1. Introduction

Surgical resection remains the cornerstone of curative treatment for early-stage and selected locally advanced non-small cell lung cancer (NSCLC) [1]. In recent years, neoadjuvant systemic therapy has undergone significant evolution with the introduction of immune checkpoint inhibitors, resulting in improved pathological response rates and event-free survival when combined with chemotherapy [2,3].

Neoadjuvant chemoimmunotherapy is established as a new standard of care in resectable NSCLC, demonstrating significantly greater rates of pathological full response compared to chemotherapy alone [3]. Furthermore, it has also been associated with improved tumour downstaging and higher rates of complete (R0) resection. KEYNOTE-671 confirmed significant improvements in event-free survival and, importantly, showed surgical feasibility, with high resection rates and no clinically meaningful increase in perioperative morbidity [4].

Notwithstanding these advancements, the relevance of neoadjuvant chemoimmunotherapy for physiologically high-risk individuals remains ambiguous [5]. Patients addressed within high-risk multidisciplinary team (MDT) routes constitute a diverse group marked by advanced age, diminished cardiopulmonary reserve, and substantial comorbidity burden. These factors are associated with increased perioperative morbidity and mortality and frequently influence treatment allocation [6,7]. Furthermore, concerns persist regarding the potential for systemic therapy-related toxicity, treatment-associated deconditioning, immune-related adverse events, and delays to definitive surgical intervention [8,9]. Such concerns may be particularly important in patients with limited physiological reserve, where even modest deterioration in functional status could compromise operability or postoperative recovery. Importantly, these patients are frequently underrepresented or excluded from major randomised clinical trials, limiting the generalisability of existing evidence and creating uncertainty regarding the optimal treatment strategy in routine clinical practice.

In contemporary practice, MDT decision-making requires a careful balancing of oncological benefits against perioperative risks [10]. While upfront surgery may be favoured in frail patients to avoid systemic therapy-related toxicity, neoadjuvant strategies may offer improved resectability and oncological clearance in those with more advanced disease [11,12]. The absence of robust, real-world data in high-risk populations creates significant uncertainty in this context and may lead to variability in treatment approaches.

Therefore, this study aims to evaluate the safety and oncological outcomes of neoadjuvant chemoimmunotherapy compared with upfront surgery in a cohort of physiologically high-risk MDT patients. Using IPTW, we sought to determine whether multimodal treatment can be delivered safely without increasing perioperative mortality, while potentially improving oncological outcomes, including rates of complete (R0) resection.

## 2. Method

### 2.1. Study Design

This was a retrospective single-centre observational study conducted at a tertiary thoracic surgical centre. Consecutive patients discussed within a dedicated high-risk multidisciplinary team (MDT) pathway between January 2022 and December 2025 who subsequently underwent surgical resection for Stage II–III NSCLC were identified from a prospectively maintained institutional database. The high-risk MDT comprised thoracic surgeons, anaesthetists and specialist nurses. All treatment recommendations were reached through multidisciplinary consensus following a comprehensive review of oncological staging, physiological assessment, operative risk, and patient suitability for multimodal treatment.

### 2.2. Patient Selection

Patients were eligible for inclusion if they met predefined criteria for referral to the dedicated high-risk multidisciplinary team (MDT). The referral criteria were developed from established high-risk thoracic surgical assessment frameworks and refined through multidisciplinary consensus among thoracic surgeons, anaesthetists, and intensive care physicians, following a review of departmental practice and outcomes. They were designed to identify patients considered at increased perioperative risk within our institution rather than to serve as a universally validated definition of high-risk.

High-risk status was determined by the presence of one or more recognised physiological, cardiopulmonary, or clinical risk factors. Inclusion criteria included performance status (PS) ≥ 2, age ≥ 80 years, forced expiratory volume in one second (FEV1) or diffusing capacity of the lung for carbon monoxide (DLCO) < 60% predicted, cardiopulmonary exercise testing (CPET) demonstrating VO_2max_ < 15 mL/kg/min, Ve/VCO_2_ slope > 35, or anaerobic threshold (AT) < 10 mL/kg/min. Additional referral criteria included shuttle walk distance < 400 m, inability to climb more than two flights of stairs, abnormal echocardiographic findings including moderate left or right ventricular dysfunction or moderate valvular disease, body mass index (BMI) < 19 kg/m^2^, anticipated requirement for pneumonectomy, significant medical comorbidities including cardiac, renal, neurological, vascular and kidney disease or technically complex cases where dual consultant operating may be required.

Patients were subsequently stratified according to treatment strategy into two cohorts: those receiving neoadjuvant chemoimmunotherapy followed by surgical resection (Chemo-IO group) and those undergoing upfront surgical resection without neoadjuvant treatment.

### 2.3. Treatment Protocol

Patients in the Chemo-IO group received platinum-based chemotherapy in combination with an immune checkpoint inhibitor according to institutional protocols and multidisciplinary treatment recommendations. The chemotherapy backbone was carboplatin-paclitaxel in 23 patients (69.7%), carboplatin-pemetrexed in 6 (18.2%), carboplatin-vinorelbine in 2 (6.1%), carboplatin-gemcitabine in 1 (3.0%), and cisplatin-vinorelbine in 1 (3.0%). Regimen selection was based on tumour histology, patient comorbidity, renal function, physiological fitness, and clinician assessment. Patients received a median of 3 treatment cycles (range 2–4). Treatment dose modification or early discontinuation was permitted in response to toxicity or intercurrent illness. Surgery was planned following radiological reassessment and multidisciplinary review.

### 2.4. Study Endpoints and Statistical Analysis

The primary endpoint was 30-day all-cause mortality following surgical resection. Secondary endpoints included 90-day mortality, complete pathological resection (R0 resection), and postoperative length of hospital stay. Mortality was defined as death occurring within the specified postoperative period, irrespective of cause. Length of stay was calculated from the date of operation until hospital discharge.

Continuous variables were summarised as mean ± standard deviation (SD) or median with interquartile range (IQR) according to data distribution. Categorical variables were presented as frequencies and percentages. Comparisons between groups were performed using appropriate parametric or non-parametric statistical tests.

To minimise confounding arising from non-random treatment allocation, inverse probability of treatment weighting (IPTW) based on propensity scores was employed [13]. Propensity scores were estimated using a multivariable logistic regression model, with receipt of neoadjuvant chemoimmunotherapy as the dependent variable. Covariates included age, sex, performance status, Charlson Comorbidity Index, forced expiratory volume in one second (FEV1), diffusing capacity of the lung for carbon monoxide (DLCO), and clinical stage.

Stabilised inverse probability weights were calculated and applied to generate a weighted pseudo-population in which baseline characteristics were balanced between treatment groups. Covariate balance was assessed using standardised mean differences (SMDs), with values below 0.1 considered indicative of acceptable balance. Weighted analyses were subsequently performed to estimate treatment effects on study outcomes. Statistical significance was defined as a two-sided *p*-value < 0.05. All analyses were performed using IBM SPSS Statistics (Version 29).

## 3. Results

### 3.1. Study Population

A total of 90 high-risk MDT patients undergoing lung resection for Stage II–III NSCLC were included in the analysis. Of these, 33 patients (36.6%) received neoadjuvant chemoimmunotherapy followed by surgery, while 57 patients (63.3%) underwent upfront surgical resection.

### 3.2. Baseline Characteristics

Baseline characteristics are summarised in Table 1. Patients receiving neoadjuvant therapy were younger than those undergoing upfront surgery (68.1 ± 7.3 vs. 71.8 ± 8.0 years, *p* = 0.027). The neoadjuvant cohort also demonstrated a significantly greater comorbidity burden, with a higher Charlson Comorbidity Index (3.2 ± 1.5 vs. 1.4 ± 1.7, *p* < 0.001).

There were no significant differences between groups with respect to sex, performance status, pulmonary function, or cardiopulmonary exercise testing parameters, including FEV1, DLCO, VO_2max_ and anaerobic threshold. Patients receiving neoadjuvant therapy were more likely to have advanced disease, with stage IIIA–IIIC tumours present in 57.6% of patients compared with 29.8% in the upfront surgery cohort (*p* = 0.014).

### 3.3. Neo-Adjuvant Treatment Characteristics

Among the 33 patients receiving neoadjuvant chemoimmunotherapy, the most commonly used chemotherapy backbone was carboplatin-paclitaxel, used in 23 patients (69.7%), followed by carboplatin-pemetrexed in six (18.2%), carboplatin-vinorelbine in two (6.1%), carboplatin-gemcitabine in one (3.0%), and cisplatin-vinorelbine in one (3.0%). Overall, 32 patients (97.0%) received a carboplatin-based regimen. The median number of treatment cycles was three (range 2–4); 23 patients (69.7%) completed three cycles, six (18.2%) completed four cycles, and four (12.1%) completed two cycles. Five patients had a documented reduction in chemotherapy dose. Reasons for treatment modification or incomplete therapy included treatment-related toxicity, acute kidney injury, thrombocytopenia, immune-related rash, and intercurrent infection. On post-treatment CT, 24 patients (72.7%) demonstrated a partial response, eight (24.2%) had stable disease, and one (3.0%) had progressive disease. All patients subsequently proceeded to surgical resection. The median interval between completion of neoadjuvant treatment and surgery was 40 days (IQR 34–47; range 26–71 days) (Table 2). 

### 3.4. Propensity Score Weighting and Covariate Balance

Given the observed baseline differences between treatment groups, IPTW was performed to minimise confounding arising from non-random treatment allocation. Propensity scores were estimated using a multivariable logistic regression model incorporating age, sex, performance status, Charlson Comorbidity Index, FEV1, DLCO and clinical stage.

Prior to weighting, patients receiving neoadjuvant chemoimmunotherapy were younger, had a greater comorbidity burden, and were more likely to present with stage III disease compared with patients undergoing upfront surgery. Following application of stabilised inverse probability weights, baseline covariates demonstrated improved balance between treatment groups. Assessment of standardised mean differences (SMDs) confirmed adequate covariate balance across all measured variables, with all post-weighting SMDs below the pre-specified threshold of 0.1.

The weighted pseudo-population, therefore, approximated a balanced comparison between treatment strategies and was used for all subsequent analyses of primary and secondary outcomes. Outcomes of the study are shown in Table 3.

### 3.5. Primary Outcome (30-Day Mortality)

Thirty-day mortality was observed in one out of 57 (1.8%) patients undergoing upfront surgery and in 0 out of 33 (0%) patients receiving neoadjuvant chemoimmunotherapy. Following IPTW adjustment, there was no evidence of increased 30-day mortality associated with neoadjuvant treatment.

### 3.6. Secondary Outcome

#### 3.6.1. Ninety-Day Mortality

Ninety-day mortality was low in both treatment groups. In the unadjusted cohort, 90-day mortality occurred in two of 57 patients (3.5%) undergoing upfront surgery and one of 33 patients (3.0%) receiving neoadjuvant chemoimmunotherapy (*p* = 1.000). Following IPTW adjustment, there remained no significant difference between treatment strategies (weighted OR 0.91, *p* = 0.948).

#### 3.6.2. R0 Resection Rate

Complete (R0) resection was achieved in 45 of 57 patients (78.9%) in the upfront surgery group and 29 of 33 patients (87.9%) in the neoadjuvant chemoimmunotherapy group. Although the neoadjuvant cohort demonstrated a higher rate of complete resection, the difference did not reach statistical significance in the unadjusted analysis (*p* = 0.394). Following IPTW adjustment, neoadjuvant chemoimmunotherapy remained associated with a numerically higher likelihood of R0 resection; however, this did not achieve statistical significance (weighted OR 3.42, *p* = 0.127).

#### 3.6.3. Length of Hospital Stay

Length of hospital stay was comparable between groups. Median length of stay was six days (IQR 4–8) in the upfront surgery cohort and six days (IQR 4–9) in the neoadjuvant chemoimmunotherapy cohort (*p* = 0.556). After IPTW adjustment, there was no significant difference in postoperative length of stay between treatment strategies (weighted mean difference 0.51 days, *p* = 0.722).

#### 3.6.4. Post-Operative Complications

Postoperative complications were generally comparable between treatment groups. Hospital-acquired pneumonia was the most frequent complication, occurring in 16 patients (28.1%) following upfront surgery and seven patients (21.2%) after neoadjuvant chemoimmunotherapy (*p* = 0.617). Acute kidney injury occurred in six (10.5%) and four (12.1%) patients, respectively (*p* = 1.000), while postoperative atrial fibrillation developed in four (7.0%) and one (3.0%) patients (*p* = 0.648). Prolonged air leak was more frequent in the neoadjuvant group (seven [21.2%] vs. one [1.8%], *p* = 0.003). Respiratory failure, pulmonary embolism, bronchopleural fistula, haemothorax, stroke, and readmission were infrequent, with no significant differences between groups (Table 4).

### 3.7. Operative Characteristics

Patients undergoing neoadjuvant therapy exhibited a higher propensity for open thoracotomy compared to those who proceeded directly to surgery (51.5% vs. 24.6%), whereas minimally invasive techniques were employed more often in the upfront surgery group (VATS: 52.6% vs. 39.4%; robotic-assisted surgery: 22.8% vs. 9.1%).

Conversion to open surgery was uncommon in both groups, occurring in 5.3% of upfront surgery cases and 9.1% of neoadjuvant cases.

Lobectomy was the predominant resection in both cohorts (87.7% vs. 81.8%). Bilobectomy was performed more frequently following neoadjuvant therapy (12.1% vs. 1.8%), whereas pneumonectomy was more commonly undertaken in the upfront surgery group (8.8% vs. 3.0%). Wedge resection was infrequent in both groups. Operative characteristics are shown in Table 5.

## 4. Discussion

This study evaluated the safety and oncological outcomes of neoadjuvant chemoimmunotherapy followed by surgery compared with upfront surgical resection in a cohort of physiologically high-risk patients with Stage II–III NSCLC discussed within a dedicated high-risk MDT. Using IPTW adjustment to minimise treatment selection bias, we found that neoadjuvant chemoimmunotherapy did not show any statistically significant difference in perioperative mortality or prolonged hospitalisation despite being administered to patients with a greater burden of comorbidity and more advanced disease. Furthermore, a numerical improvement in R0 resection rates was observed following neoadjuvant treatment.

The principal finding of this study is that neoadjuvant chemoimmunotherapy appears safe in carefully selected high-risk surgical patients. Thirty-day mortality was 0% in the neoadjuvant cohort and remained comparable to upfront surgery after adjustment for baseline differences. Similarly, 90-day mortality and postoperative length of stay did not show significant differences between treatment strategies. These findings are clinically important because concerns regarding treatment-related toxicity and delayed surgery often influence MDT decision-making in frail patients with limited physiological reserve.

Our findings are consistent with those of the landmark prospective trials CheckMate-816 and KEYNOTE-671, which demonstrated that the addition of immune checkpoint inhibitors to platinum-based neoadjuvant chemotherapy improved pathological response without compromising surgical feasibility or increasing perioperative morbidity [3,4]. These observations are further supported by recent real-world multicentre studies from Europe and England by Brunelli et al., which reported that neoadjuvant nivolumab combined with platinum-based chemotherapy could be delivered safely with acceptable perioperative outcomes and high resection rates in routine clinical practice [14,15]. Similarly, contemporary systematic reviews and meta-analyses have confirmed that neoadjuvant chemoimmunotherapy is associated with favourable pathological response rates and does not substantially increase perioperative complications compared with conventional treatment strategies [11]. However, these prospective trials and larger observational cohorts predominantly enrolled physiologically fitter patients and included relatively few individuals with severe cardiopulmonary impairment or significant medical comorbidity. Our study complements these existing data by specifically evaluating patients discussed within a dedicated high-risk multidisciplinary team, providing real-world evidence supporting the feasibility of multimodal treatment in a population that is frequently underrepresented in clinical trials.

The higher incidence of prolonged air leak (PAL) observed in the Chemo-IO group warrants careful interpretation. Although PAL occurred more frequently following neoadjuvant chemo-immunotherapy, this did not translate into a significantly longer postoperative hospital stay or increased short-term mortality, suggesting that the complication was generally manageable within contemporary perioperative pathways. Several mechanisms may contribute to the increased incidence of PAL after chemo-immunotherapy. Immune-mediated inflammatory changes and treatment-related fibrosis may alter tissue planes and increase parenchymal fragility, making pulmonary dissection and fissure division more technically demanding [16].

An additional observation from the present study was that patients receiving neoadjuvant chemoimmunotherapy underwent open thoracotomy more frequently than those managed with upfront surgery. Similar findings have been reported in other real-world series, in which a substantial proportion of patients undergoing surgery after neoadjuvant chemoimmunotherapy required thoracotomy or conversion from minimally invasive surgery [17]. This likely reflects the greater proportion of stage III disease and more anatomically complex tumours within the neoadjuvant cohort rather than a direct consequence of treatment itself. Previous studies have suggested that neoadjuvant immunotherapy may induce varying degrees of fibrosis and nodal inflammation, potentially increasing technical complexity during resection [18,19]. Nevertheless, conversion to open surgery remained uncommon in both groups, and perioperative outcomes were comparable despite a higher utilisation of thoracotomy in the neoadjuvant cohort. These findings suggest that, when performed within experienced thoracic surgical units, neoadjuvant chemoimmunotherapy does not appear to compromise surgical feasibility, even among patients considered physiologically high-risk.

Despite lacking statistical significance, on average, individuals receiving neoadjuvant chemoimmunotherapy had higher rates of complete (R0) resection in comparison to those subjected to upfront surgery (87.9% versus 78.9%). The trend favouring neoadjuvant treatment persisted after weighting, yielding a weighted odds ratio of 3.42. While the study was underpowered to detect a significant difference, this finding aligns with previous evidence demonstrating improved tumour downstaging and surgical clearance following neoadjuvant immunotherapy-based treatment.

Although inverse probability of treatment weighting (IPTW) was used to minimise confounding arising from non-random treatment allocation by balancing measured baseline characteristics, this approach cannot account for unmeasured confounders. Treatment allocation in this retrospective cohort was likely influenced by additional clinical factors that were not captured within the propensity score model, including frailty beyond performance status, nutritional status, detailed cardiopulmonary reserve, specific patterns of comorbidity, clinician judgement regarding tolerance of neoadjuvant therapy, and patient preference. Consequently, while post-weighting standardised mean differences below 0.1 indicate good balance across measured covariates, they do not exclude the possibility of residual confounding from unmeasured variables. This limitation is particularly relevant given the relatively small sample size and single-centre design, and therefore, the observed associations should be interpreted as hypothesis-generating rather than establishing causality. Larger prospective multicentre studies with more comprehensive assessment of patient fitness and treatment selection factors are required to validate these findings.

The management of high-risk NSCLC patients presents a complex clinical challenge, requiring careful balancing of operative risk against oncological benefit. In practice, there may be a tendency to prioritise upfront surgery in physiologically compromised patients to avoid the perceived risks of systemic therapy. However, the findings of this study suggest that neoadjuvant chemoimmunotherapy can be considered a feasible option in selected high-risk patients without compromising perioperative 90-day post-surgery outcomes. Furthermore, the observed improvement in R0 resection rates highlights the potential oncological advantage of a multimodal approach in this setting. These results support a more individualised MDT.

## 5. Limitations

This study is limited by its retrospective single-centre design and relatively small sample size. The high-risk MDT referral criteria represent a pragmatic institutional framework developed through multidisciplinary consensus rather than a universally validated classification. Consequently, the specific referral thresholds may not be directly applicable to all centres, although they reflect routine clinical practice within our specialist thoracic surgery service.

There is a potential for residual confounding despite adjustment for measured baseline differences using inverse probability of treatment weighting (IPTW). Although IPTW improved balance across observed covariates, unmeasured confounding cannot be excluded. Furthermore, the study was not powered to detect differences in oncological endpoints, such as R0 resection. The small number of incomplete resections resulted in imprecise effect estimates with wide confidence intervals. Consequently, the observed numerical difference in R0 resection rates should be considered exploratory and hypothesis-generating rather than confirmatory.

Nevertheless, this study provides important real-world evidence in a physiologically high-risk population that is frequently underrepresented in contemporary neoadjuvant chemoimmunotherapy trials. Larger prospective multicentre studies with longer follow-up are required to validate these findings and determine whether the observed trends translate into meaningful improvements in oncological and long-term clinical outcomes.

## 6. Conclusions

Neoadjuvant chemoimmunotherapy appears safe and feasible in high-risk surgical patients with resectable Stage II–III NSCLC. Despite being administered to patients with a greater burden of comorbidity and more advanced disease, short-term perioperative outcomes, including 30-day mortality, 90-day mortality, and postoperative length of stay, were not adversely affected when compared with upfront surgical resection. A numerically higher R0 resection rate was observed in the neoadjuvant cohort; however, this difference was not statistically significant and should be interpreted as exploratory. Larger prospective multicentre studies are required to determine whether neoadjuvant chemoimmunotherapy confers meaningful improvements in oncological outcomes, including complete resection rates.

These findings provide important real-world evidence supporting the use of multimodal treatment strategies within dedicated high-risk MDT pathways. In NSCLS stage II-III patients, physiological risk alone should not necessarily preclude consideration of neoadjuvant chemoimmunotherapy when clinically indicated. Rather, treatment decisions should incorporate both patient fitness and tumour characteristics to optimise oncological outcomes while maintaining acceptable perioperative risk.

Further multicentre studies with larger patient cohorts and longer follow-up are required to determine whether the favourable perioperative outcomes observed in this study translate into improved long-term survival and disease control. Nevertheless, the present findings support the growing role of neoadjuvant chemoimmunotherapy as part of an individualised treatment strategy for high-risk patients with resectable NSCLC.

## Figures and Tables

**Table 1:** Baseline characteristics of high-risk MDT patients undergoing upfront surgery or neoadjuvant chemoimmunotherapy prior to propensity score matching.

Variable	Upfront Surgery (*n* = 57)	Neoadjuvant (*n* = 33)	*p*-Value
Demographics			
Age at referral (years)	71.8 ± 8.0	68.1 ± 7.3	0.027
Female sex, *n* (%)	32 (56.1%)	14 (42.4%)	0.275
Comorbidity & Performance			
Performance status	0.7 ± 0.6	0.6 ± 0.6	0.334
Charlson Comorbidity Index	1.4 ± 1.7	3.2 ± 1.5	<0.001
Pulmonary function & CPET			
FEV1 (%)	86.3 ± 18.1	82.2 ± 19.1	0.322
DLCO (%)	72.7 ± 20.8	74.7 ± 17.0	0.638
VO_2max_ (mL/kg/min)	15.6 ± 3.4	16.0 ± 4.7	0.766
Anaerobic threshold (AT)	12.8 ± 3.6	13.0 ± 3.8	0.876
Oncological characteristics			
Stage IIA–IIB, *n* (%)	40 (70.2%)	14 (42.4%)	0.014
Stage IIIA–IIIC, *n* (%)	17 (29.8%)	19 (57.6%)	0.014

**Table 2:** Neoadjuvant treatment characteristics.

Characteristic	Neoadjuvant Cohort, *n* = 33
Carboplatin-paclitaxel	23 (69.7%)
Carboplatin-pemetrexed	6 (18.2%)
Carboplatin-vinorelbine	2 (6.1%)
Carboplatin-gemcitabine	1 (3.0%)
Cisplatin-vinorelbine	1 (3.0%)
Treatment cycles, median (range)	3 (2–4)
2 cycles	4 (12.1%)
3 cycles	23 (69.7%)
4 cycles	6 (18.2%)
Documented dose reduction	5 (15.2%)
Partial radiological response	24 (72.7%)
Stable disease	8 (24.2%)
Progressive disease	1 (3.0%)
Proceeded to surgery	33 (100%)
Treatment-to-surgery interval, median (IQR), days	40 (34–47)

**Table 3:** Study outcome.

Outcome	Upfront Surgery (*n* = 57)	Chemo-IO + Surgery (*n* = 33)	Unadjusted *p* Value	IPTW Effect Estimate (95% CI)	IPTW *p* Value
Primary					
30-day mortality	1 (1.8%)	0 (0%)	1.000		
Secondary					
90-day mortality	2 (3.5%)	1 (3.0%)	1.000	OR 0.91 (0.05–15.39)	0.948
R0 resection	45 (78.9%)	29 (87.9%)	0.394	OR 3.42 (0.71–16.59)	0.127
Length of stay (days)	6 (4–8)	6 (4–9)	0.556	MD 0.51 (−2.31–3.33)	0.722

**Table 4:** Post-op complications (unmatched).

Complication	Upfront Surgery (*n* = 57)	Neoadjuvant (*n* = 33)	*p*-Value
Respiratory failure	1 (1.8%)	0 (0%)	1.000
Pulmonary embolism	1 (1.8%)	0 (0%)	1.000
ARDS	0 (0%)	0 (0%)	1.000
Bronchopleural fistula	1 (1.8%)	0 (0%)	1.000
Haemothorax	2 (3.5%)	1 (3.0%)	1.000
Stroke/TIA	0 (0%)	0 (0%)	1.000
Atrial fibrillation/arrhythmia	4 (7.0%)	1 (3.0%)	0.648
Prolonged air leak	1 (1.8%)	7 (21.2%)	**0.003**
Readmission	2 (3.5%)	3 (9.1%)	0.352
Acute kidney injury	6 (10.5%)	4 (12.1%)	1.000
Hospital-acquired pneumonia	16 (28.1%)	7 (21.2%)	0.617

**Table 5:** Operative characteristics.

	Upfront Surgery (*n* = 57)	Neoadjuvant (*n* = 33)
Surgical access		
VATS	30 (52.6%)	13 (39.4%)
Open thoracotomy	14 (24.6%)	17 (51.5%)
Robotic/RATS	13 (22.8%)	3 (9.1%)
Conversion		
Conversion to open, *n* (%)	3 (5.3%)	3 (9.1%)
Resection type		
Lobectomy	50 (87.7%)	27 (81.8%)
Bilobectomy	1 (1.8%)	4 (12.1%)
Pneumonectomy	5 (8.8%)	1 (3.0%)
Wedge resection	1 (1.8%)	1 (3.0%)

## Data Availability

The original contributions presented in this study are included in the article. Further inquiries can be directed to the corresponding author(s).
